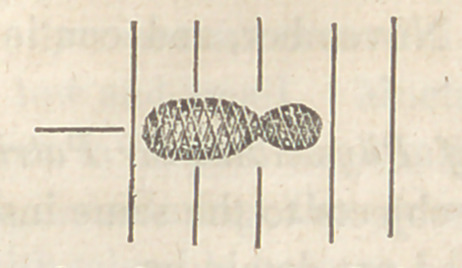# Remarks on the Heart in Its Normal Condition—Its Situation, Size, Weight, Sounds, and Their Causes, Etc.

**Published:** 1839-08-24

**Authors:** C. W. Pennock

**Affiliations:** Physician to the Philadelphia Hospital, Blockley


### MEDICAL EXAMINER.

DEVOTED TO MEDICINE, SURGERY, AND THE COLLATERAL SCIENCES.

No. 34.] PHILADELPHIA, SATURDAY, AUGUST 24, 1839. [Vol. II.

*Remarks on the Heart in its normal condition—its situation, size, weight, sounds, and their causes, etc.* By C. W. Pennock, M. D., Physician to the Philadelphia Hospital, Blockley.

To the Editors of the Medical Examiner.

Gentlemen,—Intending to communicate, in a future number of the Examiner, several cases of disease of the heart, I have thought it proper, for the more perfect understanding of the physical signs upon which the diagnosis of cardiac diseases is founded, to present a few preliminary remarks, respecting the heart in its normal condition.

Yours, etc. C. W. Pennock.

Previous to entering upon the subject of the diseases of the heart, it is of great importance that we should be acquainted with the exact situation of that organ, with that of its valves, the normal thickness of its walls, the dimensions of its several compartments, and its relative size to that of the whole frame. The object of this communication will be, therefore, briefly to indicate these several facts, and to present some views respecting the causes of the normal sounds of the heart—a subject which, for some years past, has claimed great attention from European pathologists, but which, in this country, has not been properly appreciated.

First, of the situation of the heart. If a vertical line be drawn from the second rib along the left margin of the sternum, one-third of the heart, consisting of the right auricle and one-third of the upper portion of the right ventricle, will be observed to be on the right; and two-thirds, composed of the lower portion of the right ventricle, and the whole of the left section of the heart, will be seen on the left. The apex beats two inches below the left nipple, and one inch on the sternal side. A needle passed into the chest at the upper edge of the cartilage of the fifth rib close to its costal extremity, enters the septum of the ventricles. The point of the heart is about an inch and a half below this, and to the left.

Nearly the whole of the anterior surface of the heart is formed by the right auricle and ventricle—a small portion only of the left ventricle, and its point, form part of that surface. The left auricle, and greater portion of the left ventricle, are posterior.

The lungs overlap the base of the heart, and, receding from each other, leave a portion of the heart, from one and a half to two inches square, in immediate contact with the sternum. This space, upon percussion, yields a flat sound; the remaining portion of the heart which is covered by the lungs, may be accurately determined by percussion, inasmuch as a solid body, situated beneath one which is resonant, modifies the degree of resonance of the latter. In order to measure the extent of the heart, strong percussion between the ribs, over the præcordial region, is necessary; and the preferable mode of procedure is, to commence the percussion upon the sternum, where the sound is flat, and continue it laterally to the left, until the resonance becomes distinct. In numerous instances, we have measured the heart in this way, and needles forced into the chest at the points where dulness of sound ceased, have shown that the measurement was rigidly accurate.

As the heart is attached only at the origin of the great vessels, the body and apex are free and moveable, and are influenced by the motions of the diaphragm, or by the distention of the abdomen; hence, it is important, that the fixed points of the aortic and pulmonary valves should be accurately known. To determine the precise situation of these valves, we have repeatedly forced long needles (from six to eight inches in length) into given points of the chest, perpendicular to the plane of the thorax. We have thus ascertained that the *semilunar valves of the aorta* lie immediately under the centre of the sternum, opposite the junction of the cartilage of the third ribs with that bone; *the pulmonary valves* are one-fourth of an inch to the left, at the lower edge of the cartilage of the second rib.

The aorta, from its origin, inclines in its course upwards, towards the right side of the sternum, in which direction it extends seven-eighths of an inch from the middle line of that bone. The right border of the pulmonary artery corresponds with the middle of the sternum, opposite the upper edge of the second rib; it then inclines to the left, passes around the aorta, filling the concavity of its arch; at its exit from beneath this vessel, its upper border is half an inch to the left of the middle of the sternum.

From the circumstance of the body of the heart being moveable, the mitral and tricuspid valves are not uniformly found in the same position. In several instances in normal hearts, we have found the *mitral valves* at the lower margin of the cartilage of the third rib, one-half of an inch to the left of the sternum; the *tricuspid valve* has been frequently observed under the sternum, opposite the cartilages of the fourth ribs. These points may be considered as the situations of the auriculo-ventricular openings, when the body is in a recumbent posture, and at the moment of expiration; when the individual is standing, or when inspiration takes place, the situation of these valves, in relation to the parietes of the thorax, will be changed.

*Size of the heart.* Lænnec considered it impossible to arrive at any great accuracy in the measurement of the size of this organ; he gave, as an approximation to its dimensions, that of the closed fist of the individual. This, for practical purposes, is very useful, but the measures of the heart by M. Bouillaud are much more accurate. My measurements of the heart have coincided nearly with those of Monsieur B. I will therefore give all his measures, except those of the right and left ventricles, which are slightly greater than mine. The following is the average of the measurement of the normal heart, agreeably to the author just mentioned.

Circumference measured at the base of the ventricles, 9 inches.

Length of the heart, from the origin of the aorta to the apex, 3 inches 10½ lines.

Breadth of the heart, from one border to the other, immediately below the base of the ventricles, 3 inches 11 lines.

It will be here observed, that the length and breadth of the heart are very nearly equal.

Thickness of the heart, represented by a line passing from the anterior to the posterior surface at its base, 2 inches 1 line.

*Measurement of the thickness of the several walls of the heart.*

Thickness of the left ventricle at its base, 7 lines.

Thickness of the right ventricle at its base, 2 4-5 lines.

These two last measurements are greater than I have usually found in healthy hearts. Six and a half lines for the measurement of the thickness of the left ventricle, and two lines for that of the right, are, I should say, more exact, (always excluding the columnæ carneæ.) The relative thickness of the ventricles, in general terms, may be stated as one to three.

*Measurement of the auricles.*

Thickness of left auricle, 1 3-5 lines.

Thickness of right auricle, 1 1-10 lines.

*Measurement of the circumference of the orifices of the different valves.*

Circumference of the mitral valve, 3 inches 9 lines.

Circumference of the tricuspid valve, 4 inches 1 line.

Circumference of aortic valves, 2 inches 6½ lines.

Circumference of pulmonary valves, 2 inches 10 lines.

From the above, it will be seen that the orifice of the tricuspid valve is greater than the mitral, and that that of the pulmonary valve exceeds the aortic; often, however, this difference is not found.

The circumference of the valves having been stated, the diameters of the orifices are readily ascertained: in order to conclude this part of the subject, it is only necessary to give the

*Width of the valves.*

Width of the tricuspid valve, 9⅔ lines.

This valve, in its healthy state, is very thin—thinner than fine India paper.

Width of the mitral valve, 8⅔ lines.

This valve is two-thirds thicker than the tricuspid, being equal to the thickness of thin letter paper.

Width of the semi-lunar valves of the pulmonary artery, 6 lines.

Width of the semi-lunar valves of the aorta, 6⅓ lines.

These valves frequently are precisely equal—the valves are transparent; those of the pulmonary artery are thinner and more delicate than those of the aorta.

*Capacity of the ventricles and auricles.*

The capacity of the several compartments of the heart are nearly equal; those of the right side of the heart are, however, rather greater than those of the left. In general terms, the different cavities of the heart would hold a hen's egg of ordinary size.

*Weight, bulk, and relative size of the Heart.*

Dr. Clendenning has investigated these subjects with great zeal and industry. The field of his research has been more ample than that of his predecessors; and after examining and weighing nearly four hundred hearts, he gives the following result of his observations:—"The normal heart may be assumed to average for the whole life, above puberty, about 9 oz. in absolute weight, and 8½ oz. in bulk, for the male; and 8 oz. or a little more in weight, and 7½ oz. or a little more in bulk, for the female; and to bear after death to the person, for the male, the rates of about 1 to 160, and for the female, of 1 to 150."*

*Sounds of the Heart.*

We now arrive at one of the most important and interesting questions in physiology, namely, the causes of the sounds of the heart.

Upon applying the ear to the region of the heart, two successive sounds are heard, followed by an interval of silence. The first is a dull, slow sound, succeeded immediately by a short, quick one, which is followed by a short pause.

Whilst the ear is applied to the præcordial region, and the finger is placed on the radial artery, it will be found that the impulse of the heart, dull sound, and the pulsation at the wrist, are synchronous. This correspondence of movement, sound and pulse, shows that the phenomena are owing to the contraction of the ventricles. Immediately after this dull sound, a louder, clearer, quick, and abrupt sound, (the second,) is heard, unaccompanied by any impulse, and is similar to the clicking of a small valve, or the lapping of a dog. Next follows the interval of repose.

The relative time, or rhythm, occupied by these sounds, and the period of repose, is as follows: If the whole time be a second, then the impulse and first sound will be half a second.

The second, or valvular sound, one-fourth of a second, and the period of repose also a quarter of a second.

* Clendenning, Croonian Lectures for 1838.

The measure of time may be represented by this diagram. (*Williams.*)

Lænnec, who first noticed these sounds, attributed the first to the ventricular contraction; this unquestionably is true, as the impulse, this dull sound, and the pulse, are synchronous. The second, or clicking sound, was attributed by this great man to the contraction of the auricles. This explanation of the cause of the second sound was unsatisfactory, inasmuch as the auricular contraction preceded the ventricular, and consequently his theory, as regarded the second sound, was erroneous.

Every thing respecting this subject was obscure, until the brilliant experiments of Dr. Hope on the heart's action were instituted, in 1830 and 1831, and subsequently repeated by him, Dr. Williams of London, and several committees of the British Association of Physicians. The observations of these physiologists were made upon living animals of various sizes,* after they had been deprived of sensation, whilst respiration was maintained by artificial means. The chest and pericardium being laid open, they were enabled to inspect and feel the movements, and, at the same time, hear the sounds of the heart, and thus ascertain with what motions the sounds coincided. The result of these inquiries has been, the establishment of the following facts:

*First.* That before the pericardium was opened, both sounds were distinctly heard.

*Second.* Both were also distinctly heard, though the lung was interposed between the heart and ear.

*Third.* The auricles, by a vermicular and slight action, *unattended by any sound*, contract immediately before the ventricles.

*Fourth.* The ventricular contraction, impulse, pulse in the arteries near the heart, and the first or dull sound, were seen, felt, and heard, to be simultaneous.

*Fifth.* The ventricular systole was immediately followed by the diastole, during which the second, or short clear sound, occurs.

*Sixth.* Succeeding the diastole is the interval of rest, towards the conclusion of which, the auricles contract, and the same series of movements recur.

*Seventh.* During the ventricular systole, the convexity of the ventricles became depressed, bringing the apex into forcible contact with the ribs, and thus produced the impulse.

*Eighth.* The first, or dull sound, was more distinct over the bodies of the ventricles than elsewhere. This sound remained when the auricles were opened; it continued, though somewhat diminished, when the finger was forced through the auriculo-ventricular opening, or when the mitral and tricuspid valves were destroyed. Severing the heart from the arteries did not destroy the sound.

*Ninth.* About two to three inches (on the ass) up the aorta from its origin, the second sound was heard, (but not the first,) alternating with the impulse as felt on the ventricles. It was decidedly more distinct over the origin of the aorta and pulmonary artery, than on the body of the ventricles. When the aorta and pulmonary arteries were compressed between the fingers, the first sound was accompanied by a loud murmur, and the second was stopped. When a dissecting hook was passed into the pulmonary artery, so as to prevent the closure of the semi-lunar valves, the second sound was impaired, and a hissing murmur accompanied it. A hook having been passed into the aorta, so as to prevent closure of its valves, the second sound was replaced by a prolonged hissing. Upon withdrawing the hooks, the second sound returned, and the hissing ceased.

From these experiments, the following inferences may be drawn:

*First.* That the contraction of the auricles have no influence in the production of the first sound.

*Second.* That the first sound of the heart depends on the muscular contraction of the ventricles, *accompanied probably* by a degree of valvular sound, caused by the closure of the mitral and tricuspid orifices.

*Third.* That the second sound is caused by the reaction of the arterial columns of blood, tightening and closing the semi-lunar valves at the ventricular diastole.

The preceding facts in respect to the relative intensity of the sounds over different portions of the heart, may be verified by ausculting the human chest;—thus, the first sound, which, in the experiments just alluded to, was heard loudest upon the body of the exposed ventricles, is also heard loudest upon ausculting the thorax over the space coinciding with the ventricle. The second sound, also, is heard more distinctly over the situation of the semi-lunar valves, opposite the cartilages of the second and third ribs, especially those of the right side, which are over the aortic valves, and along the course of the ascending aorta, than elsewhere.

It will be observed that the investigations reported, render it probable that the first sound is modified by the closure of the auriculo-ventricular valves. This opinion is strongly insisted on by Dr. Hope, who observes, that although the general character of the first sound be dull, yet, at its commencement near the base of the heart, there is a sudden, quick, and valvular sound, preceding, but lost in the dull muscular sound. This I have remarked in some cases of hypertrophy of the ventricles, where the contractions are slow, and the valves, though thickened, elastic.

The result of the previous investigation disproves several favourite theories; allusion will only be made to some few of the most popular. Among these is that of Magendie, who asserts

* The animals subjected to vivisection were principally asses, dogs, and young calves—the latter, having great tenacity of life, were considered as preferable to all other of the smaller quadrupeds.

that the first sound is occasioned by the rubbing of the heart against the sternum;—this was disproved by the fact, that this sound was clearly heard through a portion of interposed lung, after the anterior parietes of the chest had been removed.

By Mr. Carlisle, the first sound has been ascribed to the rush of blood into the great arteries: this was disproved, inasmuch as, when the auricles were laid open, the mitral and tricuspid valves destroyed, the arteries cut off, yet the first sound was still heard.

Mons. Rouanet attributes the first sound to the closure of the auriculo-ventricular valves: the closure of these valves has probably some influence in modifying the sound, but does not produce it, as its peculiar dull character exists after their destruction.

The investigations which we have just mentioned, have been carried forward by the brightest medical talent of Great Britain—by men, no less distinguished for their love of truth, than for their ardour in physiological investigation; and all their communications on the subject bear the evidence of great candour. Under such circumstances, implicit reliance may be given to their reports. As collateral evidence of the correctness of their views, the diagnosis founded on their explanation of the sounds of the heart, is generally verified by post mortem examinations.

I am aware that opposing views have been given, but the mass of evidence appears strongly in favour of the British physiologists. It may be advisable that the investigations should be repeated, and the writer indulges the hope, that, aided by some of his medical friends, he may undertake it. The attempt was made by them some time ago, but circumstances above their control prevented the completion of their design.

## Figures and Tables

**Figure f1:**